# Mucosa associated lymphoid tissue lymphoma of the colon: a case report

**DOI:** 10.1186/1757-1626-2-9316

**Published:** 2009-12-14

**Authors:** Cem Gezen, Metin Kement, Mustafa Oncel, Erhan Tuncay, Taflan Sahlepci, Serdar Alkan

**Affiliations:** 1 Department of Surgery, Kartal Education and Research Hospital, Istanbul, Turkey; 2 Department of Oncology, Kartal Education and Research Hospital Istanbul, Turkey

## Abstract

A 65-year-old man had suffered from rectal bleeding during defecation for a few weeks, admitted to our department. Laboratory findings were normal except a slight elevation in the level of alkaline phosphatase. Multiple polypoid lesions were observed in colonoscopic examination. The histological and immunochemical evaluation showed atypical lymphoid cell proliferation and lymphoepithelial lesions on the colonic mucosa, staining with CD20 (CD20 × 100). After the diagnosis had been confirmed as low grade mucosa associated lymphoid tissue lymphoma. Abdominal computed tomography revealed polypoid lesions throughout the colon and multiple milimetrics lymphadenopathies in the mesentery. The patient was treated with a chemotherapy regimen. During the follow-up, colonoscopic examination and blind biopsies were repeated in every 6 months, revealed endoscopically and pathologically normal mucosa each time. The patient is still alive without any recurrence of the disease 36 months after the diagnosis.

## Background

Mucosa associated lymphoid tissue (MALT) type lymphoma, first introduced by Isaacson and Wright, is thought to be occurred due to chronic antigenic stimulation triggered by persistent infection and/or autoimmune processes [1-3]. Although extra-gastrointestinal involvement has been rarely reported, MALT type lymphomas are most common in the stomach, followed by small intestine and then colon [4-6].

The therapeutic strategy in the presence of a gastric MALT type lymphoma has been well studied, however due to the rarity of the disease, a commonly accepted treatment regimen has not been established when it is located in the colon [7,8]. In the literature, patients with colonic MALT type lymphoma have been treated either with chemotherapy or surgically. The decision making between these two therapeutic modalities may be difficult, because no criteria has been established to help the medical staff to choose the right option. In this paper, a 65 year-old male diagnosed with colonic MALT type lymphoma was presented, for whom chemotherapy was indicated since the disease invaded the whole colon including the rectum, and sphincter saving procedures would be very difficult to be performed.

## Case presentation

**Figure 1:**
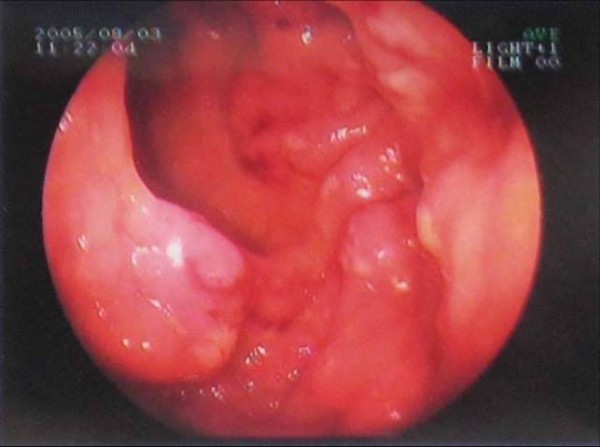
**Colonoscopy showing multiple polypoid lesions, where located closely to each other, had very thick radixes and there was almost no normal mucosa placed in-between**.

**Figure 2:**
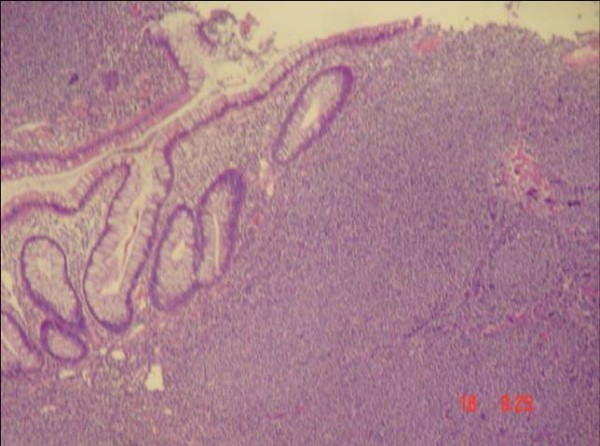
**Atypic cell proliferation in the biopsy specimens (HE × 200)**.

**Figure 3:**
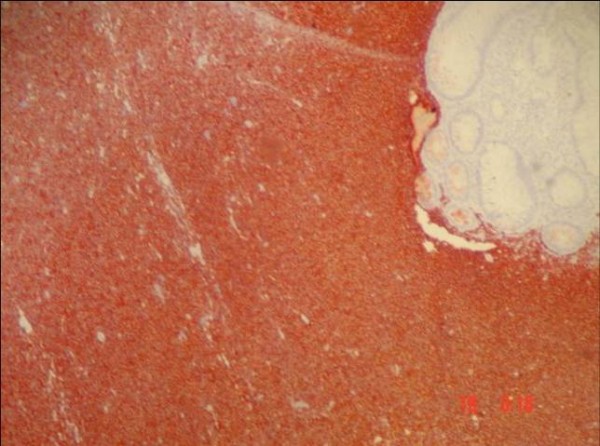
**Colonoscopic biopsy; atypic lymphoid cell strongly staining with CD20**.

**Figure 4:**
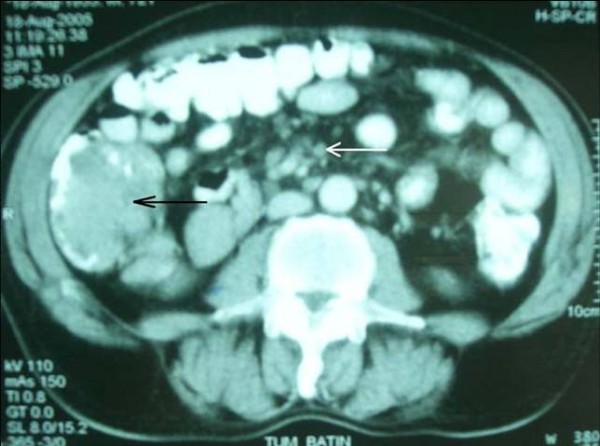
**Computerized Tomography: revealed polypoid lesion (black arrow) throughout the colon and multiple milimetrics lymphadenopathies (white arrow) in the mesentery**.

A 65-year-old Turkish man had suffered from rectal bleeding during defecation for a few weeks, admitted to our department. He denied other symptoms or signs including pain, weight loss, fatigue or enlargement of lymph nodes. Laboratory findings were normal except a slight elevation in the level of alkaline phosphatase. As further evaluation of the rectal bleeding, colonoscopic examination was performed; and multiple polypoid lesions were observed. However, the appearance of the lesions was not similar to the adenomatous polyps that were seen in patients who had polyposis syndromes. Instead, lesions were in different sizes from millimeters to a few centimeters, connected to each other without any normal mucosa in-between, and occupied the whole colon from cecum to rectum (Figure 1). Multiple biopsies were taken and the histological and immunochemical evaluation showed atypical lymphoid cell proliferation and lymphoepithelial lesions on the colonic mucosa (HE × 200) (Figure 2 and Figure 3). It was strongly staining with CD20 (CD20 × 100). After the diagnosis had been confirmed as low grade MALT lymphoma, further evaluation was indicated for stage assessment. Thorax computerized tomography (CT) was normal, however abdominal CT revealed polipoid lesions throughout the colon and multiple milimetrics lymphadenopathies in the mesentery (Figure 4).

Chemotherapy and surgical resection of the colon and rectum were the two therapeutic options. Since a sphincter-saving procedure was difficult to be performed due to the invasion of the tumor to the rectum, the patient was treated with a chemotherapy regimen consist of Cyclophosphamide 750 mg/m^2 ^(Endoxan flk, Eczacıbaşı-Baxter Istanbul, Turkey), Epirubicine 50 mg/m^2 ^(Farmorubicin fla, Carlo Erba Istanbul, Turkey), Vincristine 1.4 mg/m^2 ^(Vincristine flk., Atafarm, Istanbul, Turkey), and Prednisone 60 mg/m^2 ^(Deltacortil tb. Pfizer, Istanbul, Turkey) for 5 days in every 21 days (CHOP regimen). The regimen was continued for 6 courses, each was followed by a waiting interval of 15 days. After the therapy had been completed, a colonoscopy was performed, which was normal. In addition, the mucosa was biopsied in every 10 cm, and the pathological examination revealed the complete response of the disease to the therapy. During the follow-up, colonoscopic examination and blind biopsies were repeated in every 6 months, revealed endoscopically and pathologically normal mucosa each time. The patient is still alive without any recurrence of the disease 36 months after the diagnosis.

## Discussion

Although the characteristics of gastric MALT lymphomas have been well defined, those arising from the colon are rarely described. It has been reported that only 2.5% of all MALT type lymphomas have been identified as originated from the colon [9]. Due to rarity of the disease the controversy still continues on the diagnosis and therapy of this entity.

Schmid et al advocates that most colonic MALT lymphomas are presented as a single mass and the appearance of the lesion is generally protruding and ulcerative [10]. Contrary to this information, endoscopic examination of the current patient revealed multiple polypoid lesions located in all segments of the colon. The lesions were located closely to each other, had very thick radixes, and there was almost no normal mucosa placed in-between. This characteristic configuration can easily make one to recognize the difference between these lesions and adenomatous polyps which are observed more often. Lesions were biopsied, and the pathological examination showed atypical lymphoid cell proliferation. Since, immunohistochemical studies are generally necessitated for the accurate diagnosis, the strongly staining of the tissue with CD20 confirmed the diagnosis as low grade MALT lymphoma.

The treatment of colonic MALT lymphoma has been long debated. Because of the rarity of the disease, the data in the literature are controversial and support both surgery and chemotherapy as a first step therapy in patients with colonic MALT lymphoma. Some advocated that surgery might be the best choice since the the metastatic ability and sensitivity against chemotherapy of colonic MALT lymphoma were not known [11,12]. Similarly, Matsua et al presented another patient, had a colonic MALT lymphoma reluctant to the helicobacter pylori eradication therapy consequently underwent colonic resection [8]. Besides, different regimens of chemotherapies were successfully used for the treatment of colonic MALT lymphoma including mitoxantrone, chlorambucil, and prednisone polychemotherapy [13]. In the presented case, the whole colon was invaded with multiple lesions. Thus a sphincter-saving procedure might be difficult to be done because of the serious invasion of the rectum with the polypoid lesions. So, chemotherapy was chosen as the first step therapy and a possible surgical intervention was reserved as a second line modality only if the lesions would not respond to the chemotherapy. CHOP regimen was given for 6 courses, and consequently, the patient did not require a surgical intervention, since a complete response was observed.

Although no protocols have been established for the follow-up of these patients, the presented case repeated colonoscopies with multiple blinded biopsies were performed in every 10 centimeters after the chemotherapy had finished and once every 6 months in order to determine the compliance of the disease to the medical treatment and to find out a possible early recurrence. Complete response of the disease to the regimen was detected. No recurrence was noticed during the colonoscopic examinations and with the pathological analyses of the tissues obtained from blinded biopsies. After 36 months-long follow-up, the patient is still alive without any recurrence of the disease.

## Conclusion

Colonic MALT lymphoma is rare, however may be presented with atypical lesions and even invade the whole colon. In our opinion, chemotherapy may be the first step treatment option especially when the rectum was invaded, since to perform a sphincter-saving would be difficult in these patients. A close follow-up with colonoscopy and blinded biopsies is necessitated even if a complete response is observed.

## Consent

Written informed consent was obtained from the patient for publication of this case report and accompanying images. A copy of the written consent is available for review by the journal's Editor-in-Chief.

## Competing interests

The authors declare that they have no competing interests.

## Authors' contributions

CG involved in conception and design, analysis and interpretation of data, drafting the article, MK and MO involved in conception and design, acquisition of data. ET and SA helped in revising the article, TS involved in conception and design. All authors read and approved the final manuscript.
